# Insegurança alimentar, meio ambiente e habitabilidade em domicílios situados nas áreas urbana e rural no Brasil

**DOI:** 10.1590/0102-311XPT089424

**Published:** 2025-02-07

**Authors:** Eloah Costa de Sant Anna Ribeiro, Aléxia Vieira de Abreu Rodrigues, Luana Teixeira Ghiggino, João Henrique Rabelo Câmara, Rosana Salles da Costa, Aline Alves Ferreira

**Affiliations:** 1 Instituto de Nutrição Josué de Castro, Universidade Federal do Rio de Janeiro, Rio de Janeiro, Brasil.

**Keywords:** Saneamento Básico, Insegurança Alimentar, Urbanização, Basic Sanitation, Food Insecurity, Urbanization, Saneamiento Básico, Inseguridad Alimentaria, Urbanización

## Abstract

O objetivo deste estudo foi investigar a relação da insegurança alimentar e as condições de saneamento básico e habitabilidade em domicílios, segundo área urbana e rural no Brasil. Estudo transversal, baseado nos microdados da *Pesquisa de Orçamentos Familiares* de 2017/2018. Foram analisadas as condições de habitação a partir do tipo de acesso à água, ao escoamento sanitário, ao serviço de coleta de lixo, presença de rio, lago, baías poluídas próximas ao domicílio, presença de encosta próxima ao domicílio e presença de animais sinantrópicos. A insegurança alimentar foi aferida pela *Escala Brasileira de Insegurança Alimentar*, e considerou-se como desfecho a ocorrência dos níveis mais severos nos domicílios (insegurança alimentar moderada/grave). As estimativas de prevalência e *odds ratio* foram geradas com intervalos de 95% de confiança, e utilizou-se o software Stata. Na área urbana, os domicílios que tiveram maior chance de insegurança alimentar moderada/grave foram associados a presença de poço, descarte inadequado de lixo e falta de esgoto tratado, e na área rural, as maiores vulnerabilidades estavam relacionadas à presença de esgoto ligado à vala e ao risco de deslizamento/encosta/inundação. O saneamento básico e a habitabilidade apresentaram-se heterogêneos nas áreas rural e urbana do país, e essas condições representam desafios na garantia da segurança alimentar e nutricional.

## Abstract (en)

This study aimed to investigate the relation between food insecurity and basic sanitation and habitability conditions in households according to urban and rural areas in Brazil. This cross-sectional study was based on microdata from the 2017-2018 *Brazilian Household Budget Survey*. Habitability conditions were analyzed based on water access; sanitary drainage; garbage collection service; presence of river, lake, polluted bays near the home; presence of a slope near the home; and presence of synanthropes. Food insecurity was measured by the *Brazilian Food Insecurity Scale*. The occurrence of the most severe levels in households (moderate/severe food insecurity) was considered as the outcome. Prevalence estimates and odds ratios were generated with 95% confidence intervals on the Stata software. Urban households with higher odds of moderate/severe food insecurity were associated with wells, inadequate garbage disposal, and lack of treated sewage, whereas the greatest vulnerabilities in rural households were related to sewage connected to ditches and the risk of landslides/hills/flooding. Urban and rural Brazilians areas showed heterogeneous basic sanitation and habitability conditions, representing challenges to the guarantee of food and nutritional security.

## Resumen

El objetivo de este estudio fue identificar la relación entre la inseguridad alimentaria y las condiciones de saneamiento básico y habitabilidad en los hogares, según las zonas urbana y rural de Brasil. Estudio transversal basado en microdatos de la *Encuesta de Presupuestos Familiares* de 2017/2018. Se analizaron las condiciones de vivienda en cuanto al tipo de acceso al agua; drenaje sanitario; servicio de recolección de basura; presencia de río, lago, bahías contaminadas cerca de la casa; presencia de talud cerca de la casa; y presencia de animales sinantrópicos. La *Escala Brasileña de Inseguridad Alimentaria* se utilizó para medir la inseguridad alimentaria, y se consideró como resultado la ocurrencia de los niveles más graves en los hogares (inseguridad alimentaria moderada/grave). Se generaron estimaciones de prevalencia y razón de probabilidades con intervalos de 95% de confianza y se utilizó el software Stata. En las zonas urbanas, los hogares con mayor probabilidad de inseguridad alimentaria moderada/grave tenían la presencia de un pozo, la eliminación inadecuada de residuos y la falta de tratamiento de aguas residuales; y en las zonas rurales, las mayores vulnerabilidades se relacionaron con la presencia de aguas residuales conectadas a la zanja y el riesgo de deslizamiento de tierras/inundación. El saneamiento básico y la habitabilidad fueron heterogéneos en las zonas rurales y urbanas del país, y estas condiciones representan desafíos para garantizar la seguridad alimentaria y nutricional.

## Introdução

No Brasil, o *Censo Demográfico* de 2022 indicou que a forma de abastecimento de água, esgotamento e serviço de coleta do lixo nos domicílios foram considerados adequados para fins de monitoramento do Plano Nacional de Saneamento Básico (PLANSAB) ^1^. Apesar dos avanços na implementação de políticas públicas e sanitárias no país e com a implementação de um marco regulatório, as condições de saneamento básico ainda são heterogêneas e muito aquém dos níveis de qualidade e segurança adequados. As cidades com piores níveis de saneamento estão no Norte e Nordeste ^2^ e no território rural do país, onde há uma ausência ainda mais expressiva da cobertura de saneamento básico ^3^.

O crescimento desordenado e acelerado de cidades, assim como a expansão do agronegócio e a falta de investimentos do governo na área rural, têm causado uma série de violações ao direito humano à água, ao saneamento e à habitabilidade ^4^. Tem sido sinalizada uma intrínseca relação entre a falta de infraestrutura nas áreas de moradia e o menor acesso à alimentação de qualidade e em quantidade suficiente ^4^ ^,^ ^5^.

Nesse sentido, a insegurança alimentar faz parte de um conjunto de indicadores sociais que evidenciam o acesso à alimentação de qualidade e em quantidade suficiente nas famílias ^5^. Comumente, as condições de saneamento básico e de habitabilidade são fatores associados à insegurança alimentar, por estarem vinculados à maior vulnerabilidade dos domicílios rurais, mas que também são impactados pela infraestrutura das cidades ^4^ ^,^ ^6^. Portanto, o objetivo deste estudo foi investigar a relação da insegurança alimentar e as condições de saneamento básico e habitabilidade em domicílios, segundo área urbana e rural no Brasil.

## Métodos

Estudo transversal de base populacional realizado com os microdados da *Pesquisa de Orçamentos Familiares* (POF) de 2017/2018, realizada pelo Instituto Brasileiro de Geografia e Estatística (IBGE), que investigou 57.920 domicílios brasileiros. Detalhes sobre o plano amostral da POF 2017/2018 podem ser obtidos em relatórios já publicizados do IBGE ^7^. A POF 2017/2018 incluiu sete módulos de dados, e este estudo utilizou informações do módulo ^6^, que trata da avaliação das condições de vida das famílias. A *Escala Brasileira de Insegurança Alimentar* (EBIA) foi utilizada para classificar a situação dos domicílios em segurança alimentar e níveis de insegurança alimentar (leve, moderada e grave) ^8^. A EBIA, composta por 14 perguntas dicotômicas (sim, não), é validada no país desde 2004 para uso em estudos populacionais ^8^. Neste estudo, a variável desfecho foi a ocorrência das formas mais graves de insegurança alimentar nos domicílios (insegurança alimentar moderada/grave), tratadas de forma dicotômica (sim, não) ^9^.

Foram analisadas as condições de habitação com base em: (i) rede geral de água, quando a água no domicílio era proveniente de uma rede de serviço de água encanada, poço profundo/artesiano e poço raso/cacimba/fonte/chuva; (ii) rede de geral de escoamento sanitário, quando no domicílio o descarte de água e de dejetos sanitários estava ligado diretamente a um sistema de coleta e desaguadouro geral, fossa não ligada à rede e vala/rio; (iii) serviço de coleta de lixo, quando o lixo do domicílio era coletado diretamente pelo serviço ou por empresa de limpeza, queimado e enterrado/jogado em terreno baldio; (iv) presença de rio, lago, baías poluídas próximas ao domicílio; (v) presença de encosta próxima ao domicílio; e (vi) presença de animais sinantrópicos ^10^.

A análise estatística foi realizada considerando o desenho amostral complexo e nível de 95% de confiança (intervalo de 95% de confiança - IC95%). Utilizou-se o modelo de regressão logística para verificar a razão de chances (OR) para estimar a associação das condições de habitação com a ocorrência de insegurança alimentar moderada/grave. O modelo final considerou o ajuste pelas variáveis que estiveram associadas ao modelo bivariado (região, renda familiar *per capita* e benefícios sociais no domicílio; valor de p < 0,05).

As análises foram realizadas no software Stata, versão 16.0 (https://www.stata.com), com o comando *Survey Data Analysis* (prefixo *svy*). O estudo não necessita de aprovação em Comitê de Ética em Pesquisa, por se tratar de dados secundários de domínio público ^11^.

## Resultados

Na área urbana, a água proveniente de poço profundo estava presente em menos de 5% dos domicílios; 25% tinham escoamento sanitário por meio de fossa não ligada à rede; e 56,1% apresentavam animais vetores de doenças. Na área rural, a maioria das famílias tinha acesso à água por poço raso (40,8%), esgoto com fossa não ligada à rede (83,1%) e 53,9% realizavam queimada no lixo (Tabela 1).

**Tabela 1:** Prevalência do acesso aos serviços de saneamento básico e condições de habitação nos domicílios do Brasil segundo a área urbana e área rural. *Pesquisa de Orçamentos Familiares* de 2017/2018.

Variáveis	Brasil	Área urbana	Área rural
	% (IC95%)	% (IC95%)	% (IC95%)
Tipo de acesso à água			
Rede geral	84,9 (84,3-85,5)	93,3 (92,8-93,8)	32,2 (29,2-35,4)
Poço profundo/Artesiano	7,3 (6,8-7,7)	4,1 (3,8-4,5)	26,9 (24,8-29,2)
Poço raso/Cacimba/Fonte/Chuva	7,8 (7,4-8,3)	2,6 (2,3-2,9)	40,8 (38,2-43,5)
Tipo de acesso ao escoamento sanitário			
Rede geral	63,8 (62,9-64,7)	72,6 (71,7-73,6)	5,4 (4,0-7,3)
Fossa não ligada à rede	32,6 (31,8-33,5)	25,0 (24,1-25,9)	83,1 (81,0-85,0)
Vala/Rio	3,6 (3,3-3,9)	2,4 (2,1-2,7)	11,4 (10,1-12,9)
Tipo de acesso ao serviço de coleta de lixo			
Serviço de Coleta	90,3 (89,8-90,8)	98,7 (98,5-98,9)	37,8 (34,8-40,8)
Queimada	8,1 (7,7-8,5)	0,8 (0,6-1,0)	53,9 (51,1-56,6)
Enterrado/Jogado em terreno baldio	1,6 (1,4-1,7)	0,5 (0,4-0,6)	8,3 (7,4-9,3)
Presença de rio, lago, baía poluídos			
Sim	15,2 (14,4-16,0)	15,9 (15,0-16,8)	11,1 (9,8-12,5)
Não	84,8 (84,0-85,6)	84,1 (83,2-85,0)	88,9 (87,5-90,2)
Presença de encosta próxima ao domicílio			
Sim	11,8 (11,2-12,45)	12,4 (11,7-13,1)	8,3 (7,4-9,4)
Não	88,2 (87,5-88,8)	87,6 (86,9-88,3)	91,7 (90,6-92,6)
Presença de animais sinantrópicos *			
Sim	57,7 (56,8-58,5)	56,1 (55,2-57,1)	67,1 (65,3-68,8)
Não	42,3 (41,5-43,2)	43,8 (42,9-44,8)	32,9 (31,2-34,6)

IC95%: intervalo de 95% de confiança.

* São aqueles que se adaptaram a viver com a presença dos homens, se estabelecendo e usufruindo das condições criadas por eles, independente da vontade do homem, obtendo vantagens em relação ao acesso a abrigo, água e alimento. São exemplos de animais sinantrópicos o rato, os pombos, as baratas e os mosquitos ^10^.

A insegurança alimentar foi maior nos domicílios urbanos que tinham poço raso (21,5%), e a prevalência era ainda maior quando havia esgoto em vala/rio (22,5%) e lixo enterrado (27,7%) ou queimado (28,2%). Em domicílios rurais, a insegurança alimentar moderada/grave apresentou percentual maior na presença de animais sinantrópicos (21,1%), proximidade de rio, lago, baía poluídos (25%), esgoto em vala/rio (26%) e encosta próximo ao domicílio (29,8%) (Tabela 2).

**Tabela 2:** Prevalência de insegurança alimentar moderada/grave segundo o acesso aos serviços de saneamento básico e condições de habitação nos domicílios do Brasil segundo a área urbana e área rural. *Pesquisa de Orçamentos Familiares* de 2017/2018.

Variáveis	Área urbana		Área rural	
	Insegurança alimentar moderada/grave		Insegurança alimentar moderada/grave	
	Sim	Não	Sim	Não
	% (IC95%)	% (IC95%)	% (IC95%)	% (IC95%)
Tipo de acesso à água				
Rede geral	11,0 (10,4-11,6)	89,0 (88,3-89,6)	17,9 (15,0-21,2)	82,1 (78,8-85,0)
Poço profundo/Artesiano	18,4 (15,1-22,3)	81,6 (77,7-84,9)	15,1 (12,2-18,4)	84,9 (81,6-87,7)
Poço raso/Cacimba/Fonte/Chuva	21,5 (18,3-25,2)	78,5 (74,8-81,7)	19,0 (16,7-21,7)	80,9 (78,3-83,3)
Tipo de acesso ao escoamento sanitário				
Rede geral	9,4 (8,7-10,1)	90,6 (89,8-91,3)	12,9 (7,2-22,1)	87,0 (77,8-92,8)
Fossa não ligada à rede	17,0 (15,8-18,2)	83,0 (81,8-84,2)	15,7 (14,0-17,6)	84,3 (82,4-86,0)
Vala/Rio	22,5 (18,3-27,4)	77,5 (72,6-81,7)	26,0 (21,2-31,6)	73,9 (68,4-78,8)
Tipo de acesso ao serviço de coleta de lixo				
Serviço de Coleta	11,3 (10,7-12,0)	88,7 (88,0-89,3)	15,5 (12,8-18,7)	84,5 (81,3-87,2)
Queimada	28,2 (20,8-37,1)	71,8 (62,9-79,2)	19,7 (17,6-22,0)	80,3 (78,0-82,4)
Enterrado/Jogado em terreno baldio	27,7 (19,3-38,1)	72,3 (61, 9-80,7)	14,6 (11,1-19,0)	85,3 (81,0-88,9)
Presença de rio, lago, baía poluídos				
Não	10,5 (9,2-11,2)	89,4 (88,8-90,1)	16,7 (15,1-18,5)	83,2 (81,4-84,9)
Sim	17,1 (15,2-19,2)	82,9 (80,8-84,8)	25,0 (19,6-31,4)	75,0 (68,6-80,4)
Presença de encosta próxima ao domicílio				
Não	10,5 (9,9-11,2)	89,4 (88,8-90,1)	16,6 (14,9-18,5)	83,4 (81,5-85,0)
Sim	18,7 (16,6-21,0)	81,3 (78,9-83,4)	29,8 (24,1-36,2)	70,2 (63,8-75,9)
Presença de animais sinantrópicos *				
Não	6,2 (5,6-6,9)	93,7 (93,0-94,4)	11,2 (9,1-13,7)	88,8 (86,3-90,9)
Sim	16,2 (15,3-17,9)	83,8 (82,8-84,2)	21,1 (19,0-23,3)	78,9 (76,7-81,0)

IC95%: intervalo de 95% de confiança.

Nota: valor de p > 0,05. Insegurança alimentar moderada/grave, reduções quantitativas de alimentos e/ou interrupção do padrão alimentar decorrente da falta de alimentos entre adultos e crianças que residem no domicílio, onde assume-se que a experiência da fome é vivida.

* São aqueles que se adaptaram a viver com a presença dos homens, se estabelecendo e usufruindo das condições criadas por eles, independente da vontade do homem, obtendo vantagens em relação ao acesso a abrigo, água e alimento. São exemplos de animais sinantrópicos o rato, os pombos, as baratas e os mosquitos ^10^.

Considerando as análises do modelo final (Tabela 3), a área urbana tinha mais chance de insegurança alimentar moderada/grave quando os domicílios continham animais sinantrópicos (OR = 2,3; IC95%: 2,1-2,5), a presença de vala/rio (OR = 2,0; IC95%: 1,7-2,4), de lixo enterrado (OR = 1,9; IC95%: 1,4-2,6) e fossa não ligada à rede (OR = 1,7; IC95% 1,6-1,9). Os domicílios com lixo queimado, água proveniente de poço raso e profundo e presença de rio poluído também foram associados à insegurança alimentar (valor de p > 0,05). Na área rural, observou-se maior chance de insegurança alimentar nos domicílios que apresentavam escoamento sanitário por vala/rio (OR = 2,2; IC95%: 1,3-3,7), proximidade de encostas (OR = 2,0; IC95%: 1,6-2,5), presença de animais sinantrópicos (OR = 1,9; IC95%: 1,6-2,3), lixo queimado (OR = 1,4; IC95%: 1,2-1,7) e presença de rio poluído (OR = 1,4; IC95%: 1,1-1,8).

**Tabela 3:** Razões de chances (OR) e intervalos de 95% de confiança (IC95%) do acesso aos serviços de saneamento básico e condições de habitação segundo a área urbana e área rural, em relação à insegurança alimentar moderada/grave. *Pesquisa de Orçamentos Familiares* de 2017/2018.

Variáveis	Área urbana	Área rural
	OR (IC95%)	OR (IC95%)
Tipo de acesso à água		
Rede geral	1,0	1,0
Poço profundo/Artesiano	1,1 (0,9-1,3)	0,8 (0,6-1,0)
Poço raso/Cacimba/Fonte/Chuva	1,3 (1,1-1,5)	0,9 (0,8-1,1)
Tipo de acesso ao escoamento sanitário		
Rede geral	1,0	1,0
Fossa não ligada à rede	1,7 (1,6-1,9)	1,2 (0,7-2,0)
Vala/Rio	2,0 (1,7-2,4)	2,2 (1,3-3,7)
Tipo de acesso ao serviço de coleta de lixo		
Serviço de Coleta	1,0	1,0
Queimada	1,6 (1,1-2,2)	1,4 (1,2-1,7)
Enterrado/Jogado em terreno baldio	1,9 (1,4-2,6)	1,0 (0,7-1,4)
Presença de rio, lago, baía poluídos		
Não	1,0	1,0
Sim	1,2 (1,1-1,4)	1,4 (1,1-1,8)
Presença de encosta próxima ao domicílio		
Não	1,0	1,0
Sim	1,7 (1,5-1,9)	2,0 (1,6-2,5)
Presença de animais sinantrópicos *		
Não	1,0	1,0
Sim	2,3 (2,1-2,5)	1,9 (1,6-2,3)

Nota: valor de p > 0,05. Ajustado por região, renda familiar *per capita* e benefícios sociais no domicílio. Insegurança alimentar moderada/grave, reduções quantitativas de alimentos e/ou interrupção do padrão alimentar decorrente da falta de alimentos entre adultos e crianças que residem no domicílio, onde assume-se que a experiência da fome é vivida.

* São aqueles que se adaptaram a viver com a presença dos homens, se estabelecendo e usufruindo das condições criadas por eles, independente da vontade do homem, obtendo vantagens em relação ao acesso a abrigo, água e alimento. São exemplos de animais sinantrópicos o rato, os pombos, as baratas e os mosquitos ^10^.

## Discussão

Os resultados deste estudo mostraram que as condições de acesso ao saneamento básico e à habitabilidade nos domicílios situados nas áreas rural e urbana do Brasil estão relacionadas à presença da insegurança alimentar. Sobretudo, a ausência desses serviços foi associada aos piores níveis de insegurança alimentar, quando, na área urbana, existia a presença de animais sinantrópicos, esgoto por vala/rio, lixo enterrado, proximidade de encostas e poço raso. Já em áreas rurais, a associação foi observada quando os domicílios eram próximos às encostas, tinham animais sinantrópicos, esgoto em vala/rio, lixo queimado, proximidade de rio, lago e baías poluídos.

Apesar de mais de duas décadas políticas e programas globais e nacionais, incluindo os Objetivos de Desenvolvimento do Milênio (ODM) e os Objetivos de Desenvolvimento Sustentável (ODS), a universalização do saneamento básico e do direito à água apresenta-se estagnada e, segundo o Relatório Luz, do ano de 2024, a maioria das metas do ODS 6, que visa assegurar a disponibilidade e gestão sustentável da água e saneamento básico para todas as pessoas, estão em retrocesso desde 2015. Essa situação pode se relacionar ao baixo financiamento do Estado, cujas iniciativas, em sua maioria, não contemplam as periferias e a área rural, ocorrendo desigualdades regionais no acesso ao saneamento básico ^5^ ^,^ ^6^ ^,^ ^12^.

O ODS 6 impacta diretamente em 9 dos 17 ODS, como o ODS 2, que objetiva acabar com a fome, alcançar a segurança alimentar e melhorar a nutrição e promover a agricultura sustentável ^12^. Nesse sentido, assim como em outros estudos internacionais, os resultados nacionais demonstraram que as condições de saneamento básico e habitabilidade podem ser incluídas como mais um dos desafios em garantir o Direito Humano à Alimentação Adequada (DHAA) ^13^.

No Brasil, apesar de a renda ser um dos principais indicadores da insegurança alimentar ^14^, existe um conjunto de ações necessárias para garantir a oferta e o acesso de alimentos para toda a população. Aponta-se que o saneamento básico e a habitabilidade estão relacionados ao nexo dos sistemas alimentares, soberania alimentar e desigualdades sociais. Existe a necessidade de articulação entre diferentes setores, como a promoção da reforma agrária, da agricultura familiar, incentivo às práticas agroecológicas, vigilância sanitária dos alimentos e abastecimento de água ^15^.

O acesso à água tratada é um direito constitucional ^16^, e, apesar da abundância de recursos hídricos no país, os dados apontaram para uma possível insegurança hídrica associada à insegurança alimentar. Por sua vez, a insegurança hídrica está correlacionada às mudanças climáticas e aos impactos na agricultura (culturas, pecuária e pesca), como também no aumento da presença de animais sinantrópicos ^17^. Esses fatores podem afetar o processo da produção dos alimentos e, consequentemente, no acesso e na disponibilidade ^15^ ^,^ ^18^.

Foi observada uma associação direta entre a água proveniente do poço raso e a insegurança alimentar na área urbana, enquanto, na área rural, a presença de poço fundo foi associada à menor chance de ocorrência desse desfecho (Tabela 3). Esses resultados podem estar relacionados à execução do Programa Cisternas, que, no ano da investigação da POF 2017/2018, beneficiava quase meio milhão de famílias rurais com um sistema de captação e armazenamento de água, auxiliando o desenvolvimento local no interior do país ^18^ ^,^ ^19^, o que pode explicar parcialmente a presença de poço estar associada a uma menor insegurança alimentar.

Tanto o esgotamento sanitário como a disposição de resíduos sólidos também estão relacionados com a insegurança alimentar moderada/grave nos domicílios, principalmente com o acesso de vala/rio e fossa não ligados à rede (Tabela 2). Figueroa-Pedraza et al. ^20^ apontam que 14,1% dos domicílios na Paraíba não tinham água tratada/mineral e 71,2% não tinham acesso à rede pública de esgoto. A inadequação do esgoto no rural impacta na poluição da água disponível, pela utilização de valas e rios. Uma das consequências é a contaminação dos alimentos e o desenvolvimento de doenças parasitárias, que podem impactar no aumento da fome e da mortalidade ^10^. Por outro lado, em famílias de áreas urbanas, a falta de rede geral associa-se ao desperdício de água e ao descarte inapropriado dos resíduos nas redes de drenagem ^21^.

A coleta de lixo na área urbana por meio da rede geral foi associada à menor insegurança alimentar, porém, no território rural, ocorreu um cenário diferente, visto que existe uma maior utilização de descarte através de queimadas. Existe um contexto multifacetado dessa situação, dado que a atitude de queimar resíduos sólidos é um crime ambiental, pois causa poluição atmosférica e impactos ambientais, assim como o ato de enterrar (exceto orgânicos) ^22^.

As condições de saneamento básico estão diretamente relacionadas à habitabilidade, pois a falta de infraestrutura adequada gera ambientes insalubres, promovendo, por exemplo, o aparecimento de animais sinantrópicos, que causam agravos à saúde, prejuízos econômicos ^10^ ^,^ ^20^ e, neste estudo, se mostrou associado à maior prevalência de insegurança alimentar.

Domicílios urbanos e rurais próximos às encostas foram associados à insegurança alimentar (Tabela 3). Nas áreas urbanas, a formação inadequada das cidades gera zonas suscetíveis a deslizamentos e enchentes, principalmente em regiões periféricas e favelas, que já enfrentam desigualdades sociais e insegurança alimentar ^4^ ^,^ ^23^. A maior insegurança alimentar em áreas rurais próximas às encostas pode estar relacionada à presença da intensa exploração do solo e à degradação do meio ambiente ^23^ ^,^ ^24^ ^,^ ^25^. O desmatamento irregular dessas áreas ^26^ impacta diretamente o manejo do solo, a frequência das práticas agrícolas e a dinâmica hídrica, afetando a cadeia de abastecimento de alimentos e, a longo prazo, a capacidade produtiva e a precificação, fatores que intensificam a insegurança alimentar ^17^.

Nesse sentido, a fim de representar um compromisso global em prol de uma sociedade mais equitativa e sustentável, os resultados do estudo dialogam com mais de quatro ODS, que estão interligados e promovem territórios mais inclusivos, seguros e sustentáveis ^27^. Os resultados ressaltam a necessidade de políticas e programas intersetoriais na prática. Na teoria, o Programa Brasil Sem Fome e a Política Nacional sobre Mudança do Clima: Plano Clima 2024-2035, bem como o monitoramento do PLANSAB, seriam capazes de monitorar essa relação da insegurança alimentar e saneamento básico ^11^ ^,^ ^28^ ^,^ ^29^.

Destaca-se o ineditismo deste estudo em âmbito nacional ao associar as condições de saneamento básico e habitabilidade à situação de insegurança alimentar segundo a área do domicílio, pois, em estudos anteriores, a maioria utilizou outros indicadores sociais como sexo, raça/cor, escolaridade e renda como exposição ao desfecho da insegurança alimentar ^30^.

O Brasil já estava numa crise política e econômica desde 2016, com impactos nos níveis de insegurança alimentar ^5^. Apesar do aumento nas prevalências de saneamento básico e níveis razoáveis de habitabilidade, ainda existe um cenário aquém das metas que impacta de forma desigual as famílias das áreas urbanas e rurais, assim como nos níveis de insegurança alimentar. Portanto, há indícios que o não acesso ao saneamento básico e à água esteja relacionado à fome, necessitando também um monitoramento da segurança hídrica, além da segurança alimentar e nutricional, em perspectivas nacional e local.

## Conclusão

As situações do saneamento básico e habitabilidade foram associadas à presença de insegurança alimentar em domicílios situados nas áreas urbanas e rurais. Em destaque, a presença de animais sinantrópicos, escoamento sanitário inadequado, descarte inadequado de lixo, proximidades das encostas e presença de poço raso tiveram as maiores chances de insegurança alimentar na área urbana. Na área rural ocorreu maior associação à insegurança alimentar quando os domicílios tinham esgoto em vala/rio, domicílios próximos às encostas, animais sinantrópicos, lixo queimado, proximidade de rio, lago e baías poluídos.

Os cenários de desigualdades sociais no país refletem diretamente nas condições de saneamento básico e habitabilidade, e esses fatores podem ser considerados indicadores importantes a serem avaliados em conjunto com as condições de insegurança alimentar. A situação de saneamento básico e habitabilidade pode auxiliar na avaliação das condições socioambientais e econômicas, bem como das condições de vida da população e o acesso à alimentação em quantidade e qualidade adequadas.

## References

[1] Ministério das Cidades Plano Nacional de Saneamento Básico (PLANSAB) - relatório de avaliação anual 2021..

[2] Instituto Trata Brasil Ranking do saneamento do Instituto Trata Brasil de 2024 (SNIS 2022)..

[3] Secretaria Nacional de Saneamento. Ministério do Desenvolvimento Regional (2021). Diagnóstico dos serviços de água e esgoto. Visão geral: ano de referência 2020.

[4] Grangeiro ELA, Ribeiro MMR, Miranda LIB (2020). Integração de políticas públicas no Brasil o caso dos setores de recursos hídricos, urbano e saneamento. Cadernos Metrópole.

[5] Salles-Costa R, Ferreira A, Mattos RA, Reichenheim ME, Pérez-Escamilla R, Bem-Lignani J (2022). National trends and disparities in severe food insecurity in Brazil between 2004 and 2018. Curr Dev Nutr.

[6] Trivellato PT, Morais DC, Lopes SO, Miguel ES, Franceschini SCC, Priore SE (2019). Insegurança alimentar e nutricional em famílias do meio rural brasileiro revisão sistemática. Ciênc Saúde Colet.

[7] Instituto Brasileiro de Geografia e Estatística (2020). Pesquisa de Orçamentos Familiares: 2017-2018. Análise da segurança alimentar no Brasil.

[8] Segall-Corrêa AM, Marin-León L, Melgar-Quiñonez H, Pérez-Escamilla R (2014). Refinement of the Brazilian Household Food Insecurity Measurement Scale recommendation for a 14-item EBIA. Rev Nutr.

[9] Ribeiro ECSA, Cherol CCS, Costa RS, Castro PCP, Ferreira AA (2023). Food insecurity and social inequalities in households headed by older people in Brazil: a secondary cross-sectional analysis of a national survey.. BMC Public Health.

[10] Instituto Brasileiro do Meio Ambiente e dos Recursos Naturais Renováveis Instrução Normativa nº 141, de 19 de dezembro de 2006..

[11] Conselho Nacional de Saúde (2013). Resolução nº 466, de 12 de dezembro de 2012.. Diário Oficial da União.

[12] Grupo de Trabalho da Sociedade Civil para a Agenda 2030 Relatório Luz 2024: avaliação da implementação dos ODS no Brasil..

[13] Adams EA, Stoler J, Adams Y (2019). Water insecurity and urban poverty in the Global South implications for health and human biology. Am J Human Biol.

[14] Lignani JB, Sichieri R, Burlandy L, Salles-Costa R (2011). Changes in food consumption among the Programa Bolsa Família participant families in Brazil. Public Health Nutr.

[15] Salles-Costa R, Ferreira AA, Castro P, Burlandy L (2022). Sistemas alimentares, fome e insegurança alimentar e nutricional no Brasil.

[16] Brasil (2007). Lei nº 11.445, de 5 de janeiro de 2007. Estabelece as diretrizes nacionais para o saneamento básico; cria o Comitê Interministerial de Saneamento Básico.. Diário Oficial da União.

[17] Brasil (2023). Decreto nº 11.550, de 5 de junho de 2023. Dispõe sobre Comitê Interministerial sobre Mudança do Clima.. Diário Oficial da União.

[18] Arsk I (2020). Os efeitos do Programa Cisternas no acesso à água no semiárido. Desenvolvimento e Meio Ambiente.

[19] Palmeira PA, Bem-Lignani J, Salles-Costa R (2022). Acesso aos benefícios e programas governamentais e insegurança alimentar nas áreas rurais e urbanas do Nordeste brasileiro. Ciênc Saúde Colet.

[20] Figueroa-Pedraza D, Alves-Bezerra T, Cerqueira ACDR, Santos-Da Fonsêca J Segurança alimentar de famílias residentes em um município do interior da Paraíba.Brasil (2017). Rev Salud. Pública.

[21] Oliveira G, Scazufca P, Sayon P Ranking do saneamento - Instituto Trata Brasil 2022 (SNIS 2020)..

[22] Brasil (1998). Lei nº 9.605, de 12 de fevereiro de 1998. Dispõe sobre as sanções penais e administrativas derivadas de condutas e atividades lesivas ao meio ambiente, e dá outras providências.. Diário Oficial da União.

[23] Landau EC, Moura L (2016). Variação geográfica do saneamento básico no Brasil em 2010: domicílios urbanos e rurais.

[24] Trivellatto G, Leme LML, Lucas A (2021). Submissão da agricultura à indústria, colapso ambiental e multifuncionalidade da agricultura no contexto brasileiro. PerCursos.

[25] Lima FV, Silvino GS, Melo RSS, Lira EC, Ribeiro TS (2015). Variabilidade espacial de atributos físicos do solo em área de encosta sob processo de degradação. Revista Caatinga.

[26] MapBiomas Relatório anual do desmatamento no Brasil..

[27] Programa das Nações Unidas para o Desenvolvimento ODS em ação..

[28] Instituto Brasileiro de Geografia e Estatística Censo Demográfico 2022. Panorama..

[29] Ministério do Desenvolvimento e Assistência Social, Família e Combate à Fome Brasil sem fome..

[30] Lignani JB, Palmeira PA, Antunes MML, Salles-Costa R (2020). Relationship between social indicators and food insecurity a systematic review. Rev Bras Epidemiol.

